# Supplementary material for: Wellbeing and coping of UK nurses, midwives and allied health professionals during COVID-19-a cross-sectional study
Source: PLoS One. 2022 Sep 21;17(9):e0274036. doi: 10.1371/journal.pone.0274036 (PMC9491587; doi:10.1371/journal.pone.0274036)
Supplement: Attachment — Submitted filename: Response to reviewers.docx [file pone.0274036.s001.docx]

**6^th^ October 2021**

**Wellbeing and Coping of UK Nurses, Midwives and Allied Health Professionals during COVID-19-a cross sectional study**

**Response to Reviewers:**

The authors would like to thank the reviewers for taking their time to consider this manuscript and for providing detailed feedback.

**Reviewers' comments:**

**Reviewer #1:** This submission is obviously relevant and timely. It is clearly-written, and clearly-reasoned throughout. The descriptive and regression statistical analyses are competently done and well elaborated in the text and tables. The chosen measures are psychometrically sound (adequate scale alphas etc.), and appropriate to the task.

A few (very minor) suggested revisions/rephrasings:

In table 2, with both unweighted and weighted results, there is too much crammed into the one table. Is it absolutely necessary to list both unweighted and weighted here? If it is, perhaps do this as two separate tables.

**Author comments:** The authors have separated the table into two (Table 2 & 3; lines-300-313).

367 coping strategies account for 36.1%, F(15, 560)=15.95, p<.001). Only six of the coping variables
368 contributed significantly to the explanation of mental wellbeing. Positive framing
((Rephrase, clarifying what is meant by 'significantly'. It's not clear whether this refers to importance or statistical significance.))
**Author comments:** The authors have added the following: “Only six of the coping variables contributed statistically significantly to the explanation of predicting positive mental wellbeing.”(line 357-358)

520 quality of working life over the two different time points and has identified//demonstrated how positive coping
521 strategies and support can//could potentially help improve workforce wellbeing. This is a strength of the cross
((I would suggest rephrasing identified/demonstrated and can/could potentially))

**Author comments:** The authors have taken the reviewers suggestions for rephrasing (lines 511-512)

**Reviewer #2:** Thank you for giving me the pleasure to review your manuscript. Overall, it is a very nice work. However, I have the following comments

**1-** My concern is how the authors made sure that the 2nd wave of data collection had the same participants as in wave 1 given that other than emails, social media advertising was used and an e-survey was used for data collection?

**Author comments:** The authors noted that the study was cross-sectional and have highlighted this within the limitations section of the discussion; “The survey is however limited by the use of the cross-sectional data collected, meaning that it is only reflective of the healthcare workforce at that current time point. Therefore, the data cannot reveal causal relationships as we cannot infer cause and effect”. (lines 515-517) Therefore due to confidentiality and different survey collection points we cannot ascertain which respondents answered the Phase 1 and Phase 2 surveys. The surveys are cross-sectional and stand-alone as there are different questions also asked in each, so it cannot be determined if the same participants completed the survey. Additionally, the same methods of data collection were used in both phases and the same data recruitment methods as noted on lines 136-148.

**2-** There is not enough description about the mental wellbeing scale used in the study? Giving some examples of the items would be beneficial

**Author comments:** Lines 180-184 have been amended to include: *“Mental wellbeing was assessed using the short version of the Short Warwick-Edinburgh Mental Wellbeing Scale (SWEMWBS) [29], a positively worded seven-item scale assessing statements about thoughts and feeling which asks respondents to describe their experiences of how often they felt this way in the last two weeks.”* This statement is a reflective description of the scale as detailed by the creators of the SWEMWS, Stewart et al. (2009).

3- The regression tables do not present the full model as sociodemographic variables were excluded from the tables although they were used as predictors.

**Author comments:** The authors have amended Tables 5 and 6 to include the sociodemographic variables (lines 341-347)

4- As the regression analyses focused on coping strategies as predictors for mental well-being and quality of working life, this should be reflected in the results section of the abstract as well and in the introduction by reporting studies that investigated coping strategies used by healthcare workers during the pandemic.

**Author comments:** The authors have amended the abstract to include the following statement: *“The COVID-19 pandemic had a significant effect on the psychological wellbeing, quality of working life which decreased while the use of negative coping and burnout of these healthcare professionals increased.”(lines 44-45).*

5- Was wondering if you thought about using coping strategies as a moderator/mediator between (mental well-being and quality of working life) and burnout as an outcome

**Author comments:** The authors did consider this but as burnout was only measured in Phase 2, a comparison could not be made. The authors decided to use coping strategies as a moderator between mental wellbeing, quality of working life and burnout in a later study comparing Phases 1, 2 and the recently completed Phase 3 data.

As per the Editorial Assistant’s email, please find the financial disclosure statement:

**Funding Statement:** The authors would like to thank the Northern Ireland Social Care Council (NISCC), and the Southern Health and Social Care Trust in Northern Ireland for seed funding, the Public Health Agency R&D Office Northern Ireland for study funding. The study is also supported by the National Institute for Health Research (NIHR) Policy Research Programme, through the Policy Research Unit in Health and Social Care Workforce, PR-PRU-1217-21202. The views expressed are those of the authors and not necessarily those of the funder or the NIHR Policy Research Programme.

Yours sincerely

Patricia Gillen

Dr Patricia Gillen

Email: [p.gillen@ulster.ac.uk](mailto:p.gillen@ulster.ac.uk)
