# Supplementary material for: Wellbeing and coping of UK nurses, midwives and allied health professionals during COVID-19-a cross-sectional study
Source: PLoS One. 2022 Sep 21;17(9):e0274036. doi: 10.1371/journal.pone.0274036 (PMC9491587; doi:10.1371/journal.pone.0274036)
Supplement: Attachment — Submitted filename: Response to reviewers.docx [file pone.0274036.s002.docx]

**12^th^ May 2022**

**Wellbeing and Coping of UK Nurses, Midwives and Allied Health Professionals during COVID-19-a cross sectional study**

**Response to reviewers comments:**

The authors would like to thank the reviewers for taking their time to consider this manuscript and for providing detailed feedback.

In addition to the reviewer comments, please address the following

Please specify whether the design is cross-sectional or longitudinal given that data was collected at two time points.

Authors’ comments: The authors would like to note that the abstract and methods state that cross-sectional data was collected at two different time points during the pandemic.
Please explain how the sample was selected for both surveys- was this a random sample? If the sample is random, the study design would be more correctly described as a repeated cross-sectional design.

Authors’ comments: The authors have now noted that a similar cross-sectional survey was repeated at different time points through a convenience sampling recruitment approach.
How did you address the possibility of a cohort effect?

Authors’ comments: To mitigate a cohort effect as mentioned by the authors in the analysis section of the manuscript, data was weighted. The authors also noted in the limitations that *“a longitudinal data collection and examination of the effects on the variables over time would enable a more detailed exploration of the relationships, however given the nature of the pandemic it would be more difficult to record this information over a long period.”*
Please provide the rationale for including burnout only in Phase Two?

Authors’ comments: The authors introduced the burnout scale into Phase 2 following on from qualitative data presented in Phase 1 of the study. The scale has since been used in the subsequent phases of the wider study (Phases 3 and 4) and is relevant to the current academic literature citing burnout amongst health and social care professionals.
Please specify the type of multiple regression that was conducted.

Authors’ comments: The authors’ have now noted that a linear regression was used.
Independent t-tests - The Independent Samples t Test compares the means of two independent groups in order to determine whether there is statistical evidence that the associated population means are significantly different. The Independent Samples t Test is a parametric test.
How did you address the chance that you will make a Type I error?
Authors’ comments: The authors’ looked to decreased the chance of Type 1 errors by adjusting the alpha level. The authors did use significant levels of 0.05 and 0.01, however issues which lower the alpha level can lead to the results of the hypothesis test not capturing the true difference in the data.

**Reviewers' comments:
Reviewer's Responses to Questions**

**Comments to the Author**
Reviewer #2: Thank you for giving me the opportunity to review this manuscript. It was obvious that a lot of work has been committed to it. However, I have the following comments

Abstract
1- The authors mentioned that their data is cross-sectional and then they mentioned that they collected data at two-time points, and this is confusing since cross-sectional means data are collected at one time period of the study.

Authors’ comments: The authors have now mentioned that this is a wider study which utilised a repeated cross-sectional design, and the data presented in this current paper were collected at two different time periods; May-July 2020 (Phase 1 of study) and November-February 2021 (Phase 2 of study).

Introduction
1- Well-written and culturally situated
2- In the study aim, the authors did not mention they also studied burnout among their sample, but in the abstract section, it was mentioned. Please be consistent

Authors’ comments: The authors have added in this into the study aims for consistency.

Design:
1- Can you please detail how the emails were obtained for participants?

Authors’ comments: The authors did not obtain any emails from the participants, no personal identifying information was obtained. “Respondents were recruited by convenience sampling through emails, newsletters and social media posts of employers, regulatory bodies, professional communications, professional associations and workplace unions. Participation in the study at each time point was voluntary and the data were collected through an online survey hosted on the Qualtrics platform.” Participants were not asked for email address, therefore while we may have recruited the same participants at each cross-sectional study we cannot confirm this.

2- Can you give an example or two of some of the SWEMWBS scale items?

Authors’ comments: Examples of the scale items are: I’ve been dealing with problems well, I’ve been feeling relaxed.
3- Why burnout was only measured in the 2nd phase of the study?

Authors’ comments: The authors introduced the burnout scale into Phase 2 following on from qualitative data presented in Phase 1 of the study. The scale has since been used in the subsequent phases of the wider study (Phases 3 and 4) and is relevant to the current academic literature citing burnout amongst health and social care professionals.

Results:
1- I’m not sure what is the point of comparing the two phases of data collection. I guess the authors may be right in that some respondents of phase one are the same in phase two, but this is just an assumption. Therefore, to say for example, quality of working life was lower in phase 2 than phase 1, this may relate to that the participants are different , not to the variable per se.

Authors’ comments: The authors’ note in the limitations section the issue with cross-section design and acknowledge that comparing two different dataset may not have had the same participants but this allows a comparison to a generalised overview of the possible trend.
2- What type of regression analysis was used?

Authors’ comments: The authors note that linear regressions were used and have added this to the manuscript.

Discussion
Saying that coping was used as an interaction with phase will let the reader understands that an interaction term was created, but this is not the case in the study. Therefore, please change the wording.

Authors’ comments: The authors note in the analysis that interaction terms were created.
- The authors mentioned “Findings in this current study highlighted a decrease in the use of positive coping strategies 430 (active coping, planning, acceptance, positive reframing) while a significant increase was evident 431 in the use of negative avoidant coping strategies (venting, self-blame, behavioral disengagement) 23 432 between Phases 1 and 2”. I am still having hard time to grasp comparing the variables between phase 1 and 2 as I mentioned before, this may relate to having a different sample in phase 2 from phase 1

Authors’ comments: The authors acknowledge generalisation cannot be made but found it important to examine the variables over multiple time periods of the COVID-19 pandemic to provide an example or note a possible trend in issues affecting this workforce during a difficult time.
- “The association of using behavioural engagement and planning greatly decreased from Phase 1 to Phase 2, which suggested a negative impact with using this strategy. As stress and coping strategies are interlinked, the continued uncertainty and increasing stressors associated with the pandemic could 450 have further detrimental effects on the wellbeing and work-related quality of life of healthcare professionals.” Comparing this paragraph with the results in the regression table, shows that planning was not a significant predictor of well-being in phase 1, but it was significant for phase

2. Then planning has been increased by looking at beta values . The same for behavioral disengagement.
Authors’ comments: The authors’ have negative beta values for the planning and behavioural disengagement.
I recommend that the authors just pick one phase of the study to report the findings and make sure that reporting and discussing the findings are congruent with the numbers in the tables.

Authors’ comments: The authors acknowledge this statement but do feel that this paper will add something to the current literature and is important to explore across the two phases. However if the reviewer feels strongly we can change this.

Reviewer #3: This is a very interesting study. The article is well organised and written and the authors have addressed the points raised by the other reviewers.
